# Urinary pentosidine as a potential biomarker of muscle and physical performance in young adult men

**DOI:** 10.1186/s40101-024-00376-1

**Published:** 2024-11-21

**Authors:** Takayuki Nishimura, Ping Yeap Loh, Yoshihito Tomita, Ted K. S. Ng, Takafumi Maeda

**Affiliations:** 1 Department of Human Life Design and Science, Faculty of Design, Kyushu University, Fukuoka, 815-8540 Japan; 2 Department of Physical Therapy, School of Rehabilitation, Tokyo Professional University of Health Science, Tokyo, Japan; 3 Rush University Medical Center, Rush Institute for Healthy Aging, Chicago, IL USA

**Keywords:** Pentosidine, Muscle strength, Ultrasound, Bone mass, Muscle thickness

## Abstract

Pentosidine is representative of the cross-linked structure of advanced glycation end products (AGEs) and has been suggested as a biomarker to assess bone and muscle quality. As studies on pentosidine in young adult men remain limited, we aimed to clarify the associations of urinary pentosidine with musculoskeletal status and physical performance in young men. Participants in this study comprised 32 men (age range: 19–39 years). Anthropometric measurements (body composition by InBody 430; stiffness index by ultrasound), muscle performance (grip strength by dynamometer, thigh muscle thickness by ultrasound), physical performance (functional reach test, 30-s chair stand test, and timed up and go test), and urinary biomarkers (pentosidine, N-telopeptide of type I collagen, and creatinine) were measured. In partial correlation analysis adjusted for age and height, higher urinary pentosidine levels were significantly associated with lower fat-free mass index (rho = − 0.368, *p* = 0.046), grip strength (rho = − 0.433, *p* = 0.017), rectus femoris thickness (rho = − 0.393, *p* = 0.032), and anterior thigh thickness (rho = − 0.416, *p* = 0.022), and a marginally inverse correlation was noted between urinary pentosidine levels and functional reach test (rho = − 0.327, *p* = 0.078). Our findings suggest that pentosidine correlates inversely with a few muscle and physical performance indicators. Pending future validations, urinary pentosidine may be a biomarker of AGEs in young men.

## Background

Peak muscle mass and peak bone mass during young adulthood are important to maintain and prevent losses of muscle mass and bone mass in late adulthood [1]. Simple and easily accessible methods of evaluation to achieve a comprehensive overview are thus required to understand the state of physiological health, such as muscle and bone health, in young individuals.

Pentosidine is a biomarker representative of cross-linked structures of advanced glycation end products (AGEs), which form following the oxidation of bone collagen cross-links [2]. Urine pentosidine levels increase with age [3], and urinary pentosidine has been reported as an independent risk factor for fracture in postmenopausal women, regardless of age and bone density [4]. A negative association between serum pentosidine level and muscle strength has also been identified in elderly men and women [5]. In contrast, handgrip strength at 12 years old negatively predicted urinary pentosidine level at 14 years old in a cohort study [6]. Urinary pentosidine may thus be a useful biomarker for assessing bone, muscle strength, and muscle mass in puberty and late life.

Urinary pentosidine may also show potential as a simple marker for evaluating muscle and bone health in young adults. Despite previous studies on older adults and adolescents, data on young adults remain limited. We aimed to clarify the associations between urinary pentosidine, musculoskeletal status, and physical performance in young men. We hypothesized that urinary pentosidine level correlates to musculoskeletal and physical performance parameters.

## Methods

Thirty-two men (age: 24.3 ± 5.4 years; range: 19–39 years) from Fukuoka city in Japan participated in this study. Data collection was conducted in August 2022. All participants provided written informed consent prior to examination. This study was approved by the Research Ethics Committee of the Faculty of Design at Kyushu University (approval no. 2022–491).

### Anthropometric measurements

Height was measured using a stadiometer (HM200P; Charder Medical, Taichung City, Taiwan). Body composition values such as body mass (BM), fat-free mass index (FFMI), and skeletal muscle mass were measured by bioelectrical impedance analysis using an InBody 430 (InBody Japan Inc., Tokyo, Japan). Muscle thicknesses of the rectus femoris and anterior thigh (quadriceps) muscle of the dominant leg were measured using a VIAMO ultrasound system (Toshiba, Tokyo, Japan) with a PLT-1204BT transducer (imaging frequency bandwidth, 12 MHz). Lastly, heel stiffness index (bone mass) was measured by quantitative ultrasound using a Lunar Achilles device (GE Lunar Corp., Madison, WI, USA).

### Muscle and physical performance tests

Four performance tests were conducted. First, grip strength of the dominant hand was measured twice using a digital dynamometer (T.K.K. 5401; Takei Scientific Instruments, Niigata, Japan) and averaged. Second, the timed up and go test (TUG), comprising getting up from a chair, walking forward 3 m, turning around, walking back to the chair, and sitting back down, was performed using a TUG measurement set (T.K.K. 5804, Takei Scientific Instruments). The task was completed by the participant at their usual walking pace, with the average of two trials taken as the TUG score. Third, the functional reach (FR) test was performed with the initial position (standing comfortably upright, facing forward, hand in a fist, arm extended) and furthest reaching point (reaching forward as far as possible without stepping or losing balance) as the 2 points measured. Scores were averaged over two attempts. Fourth, participants were instructed to complete as many full stands as possible in the 30-s chair stand test. In the 30-s chair stand test, the participant sits on a chair with feet flat, knees at 90°, and arms crossed over the chest. They stand up using only their legs, keeping the back straight, then return to a seated position in a controlled manner, complete maintaining proper form and smooth motion throughout, and then count one stand.

### Urine collection and analysis of biomarkers

For biomarker analyses, second urine samples were collected between 09:00 and 12:00, and we instructed subjects to avoid eating and drinking before experiment from 2 h ago. Urinary pentosidine levels were measured using high-performance liquid chromatography. In this study, urinary N-telopeptide of type I collagen (NTx) was also measured as it is generally related to bone mass, allowing us to compare the sensitivity of pentosidine in relation to bone mass. NTx levels were determined using chemiluminescent enzyme immunoassay. In addition, urinary cortisol concentrations were assessed using chemiluminescent immunoassay. These values were corrected to creatinine (Cre) concentrations.

### Statistical analysis

Variables are presented as the mean ± SD (standard deviation). Pearson’s correlation analysis was used to assess correlations between urinary biomarkers and other parameters. Partial correlation analysis was used to adjust for confounding factors (age and height) for correlation coefficients refer to previous study [7]. Statistical significance was set at the level of *p* < 0.05. Due to the exploratory nature of this study, no corrections for multiple testing were performed. Data were analyzed using the Statistical Analysis System OnDemand for Academics (SAS Institute, Cary, NC, USA).

## Results

The characteristics of the study population are presented in Table 1. Mean pentosidine level was 5.2 ± 1.1 pmol/ml Cre.

**Table 1:** Characteristics of the study population

	Mean	SD
Age, years	24.3	5.5
Height, cm	171.7	7.1
Weight, kg	62.7	13.3
BMI, kg/m^2^	21.5	5.2
Body fat, %	17.1	6.3
FFMI	17.4	2.0
Grip strength, kgf	40.4	6.7
Rectus femoris thickness, mm	20.7	4.3
Anterior thigh thickness, mm	39.7	9.9
Stiffness index	110.0	22.2
Functional reach test, cm	48.5	5.2
30-s chair stand, repetitions	33.2	4.6
TUG, s	4.3	0.5
Pentosidine, pmol/ml Cre	5.2	1.1
Ntx/Cre, nmol BCE/mmol Cre	45.8	14.2

*BMI* body mass index, *FFMI* fat-free mass index, *TUG* timed up and go test

**Fig. 1:**
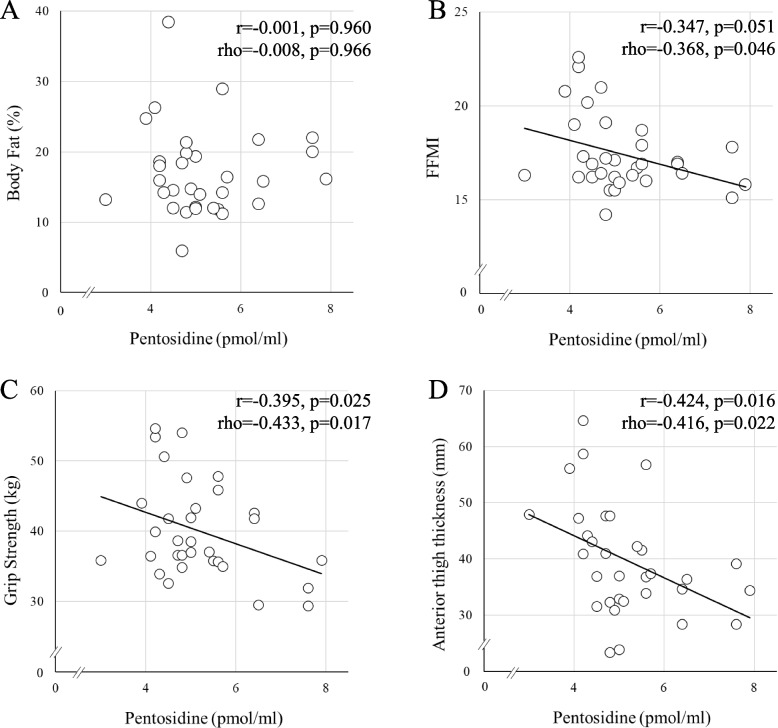
Scatter plot of pentosidine and measurements. FFMI, fat-free mass index; r, simple correlation coefficients; rho, partial correlation coefficients. Pentosidine correlates inversely with FFMI, grip strength, and anterior thigh thickness but shows no correlation with body fat. Partial correlation coefficients are adjusted by age and height

In partial correlation analysis, significant inverse correlations adjusted by age and height were found between urinary pentosidine levels and each of FFMI (rho = − 0.368, *p* = 0.046; Table 2, Fig. 1B), dominant grip strength (rho = − 0.433, *p* = 0.017; Table 2, Fig. 1C), dominant rectus femoris thickness (rho = − 0.393, *p* = 0.032; Table 2), and dominant anterior thigh thickness (rho = − 0.416, *p* = 0.022; Table 2, Fig. 1D). No significant correlation was seen between pentosidine level and body fat percentage (Table 2). A marginally inverse correlation adjusted by age and height was observed between urinary pentosidine levels and the FR test (rho = − 0.327, *p* = 0.078; Table 2).

**Table 2:** Simple and partial correlation coefficients between pentosidine and other parameters

	*r*	*p*-value	rho	*p*-value
Body fat (%)	− 0.001	0.960	− 0.008	0.966
FFMI	− 0.347	0.051	− 0.368	0.046
Grip strength (kgf)	− 0.395	0.025	− 0.433	0.017
Rectus femoris thickness (mm)	− 0.396	0.025	− 0.393	0.032
Anterior thigh thickness (mm)	− 0.424	0.016	− 0.416	0.022
Stiffness index	− 0.282	0.118	− 0.247	0.189
Functional reach test (cm)	− 0.302	0.093	− 0.327	0.078
30-s chair stand (repetitions)	0.155	0.398	0.175	0.356
TUG (s)	− 0.135	0.462	− 0.131	0.489

*FFMI* fat-free mass index, *TUG* timed up and go test, *r* simple correlation coefficients, rho partial correlation coefficients. Partial correlation coefficients were adjusted by age and height

A significant inverse correlation adjusted by age and height was observed between urinary NTx level and TUG (rho = − 0.372, *p* = 0.043; Table 3).

**Table 3:** Simple and partial correlation coefficients between Ntx/Cre and other parameters

	*r*	*p*-value	rho	*p*-value
Body fat (%)	− 0.018	0.921	0.011	0.953
FFMI	− 0.279	0.122	− 0.209	0.267
Grip strength (kgf)	− 0.173	0.343	− 0.091	0.634
Rectus femoris thickness (mm)	− 0.116	0.528	− 0.102	0.591
Anterior thigh thickness (mm)	− 0.126	0.493	− 0.140	0.462
Stiffness index	− 0.102	0.580	− 0.119	0.531
Functional reach test (cm)	− 0.057	0.756	− 0.003	0.986
30-s chair stand (repetitions)	− 0.184	0.313	− 0.156	0.412
TUG (s)	− 0.339	0.057	− 0.372	0.043

*FFMI* fat-free mass index, *TUG* timed up and go test, *r* simple correlation coefficients, rho partial correlation coefficients. Partial correlation coefficients were adjusted by age and height

## Discussion

### Associations of pentosidine with grip strength, rectus femoris thickness, and anterior thigh muscle thickness

Our results indicated inverse associations between pentosidine level and grip strength, rectus femoris muscle thickness, and anterior thigh muscle thickness. Conversely, no association with body fat percentage was observed. Grip strength and thigh muscle thicknesses are similar but represent distinct dimensions of muscular health. Grip strength is often used to reflect whole-body muscle strength and is widely linked to general health and longevity [8–10]. Similarly, thigh muscle thickness from ultrasound could indicate overall body muscle mass, which is an important component of physical fitness and mobility as seen in the older adult population [11, 12]. Our findings suggest a potential biological mechanism for links between muscle and AGEs as indicated by pentosidine levels that may potentially be explained by increased insulin resistance. Skeletal muscle tissue is the predominant site of insulin-mediated glucose uptake [13], and low skeletal muscle strength could increase blood glucose levels and lead to increased AGE production [7, 14]. Pentosidine could therefore serve as a potential biomarker for AGE accumulation and help in understanding muscle health, even in young individuals. We provide new data of the association between pentosidine and muscle strength in young people.

### Associations of pentosidine with physical performance

In this study, we observed a marginally inverse correlation between pentosidine levels and the FR test, but no significant associations with other physical performance metrics (30-s chair stand and TUG). While the underlying mechanisms of this finding remain unclear, these results suggest that lower pentosidine levels may be associated with higher physical flexibility, potentially through improved posture maintenance facilitated by higher muscle strength at lower pentosidine levels. This interpretation is preliminary and requires further investigation.

### Associations of NTx with measurement parameters

No significant correlation was seen between urinary Ntx and bone mass or other parameters, except TUG. The association between Ntx and TUG was difficult to explain, because of the unclear physiological mechanisms and inconsistent associations between parameters. While Ntx is an appropriate biomarker for evaluating bone mass and physical performance in older adults [15–17], the present results suggest it may not be suitable for young adults. Similarly, previous studies have shown no relationship between bone mass and Ntx in people under 50 years old [16, 17]. This difference seems to depend on the balance between bone turnover and formation, which decreases with aging [17]. Our results are consistent with findings from previous studies, and paradoxically, pentosidine may be a candidate biomarker for evaluating musculoskeletal status in young adults.

Previous research has shown that low muscle strength causes AGEs, including pentosidine, to increase and accumulate [5, 6, 18]. More importantly, pentosidine is associated with not only physical strength but also mental health [6]. Taken together with previous findings, these results highlight the importance of intervention. Since our findings were cross-sectional, the direction of causality cannot be ascertained. Strategies aimed at reducing the accumulation of AGEs, such as dietary modifications, increased physical activity, or other lifestyle changes, could be crucial for preserving muscle health and preventing future declines [19–21].

This study has several limitations. First, as an exploratory study, we are unable to establish a causal relationship due to the study design. Second, the small sample size and limited measurement parameters make it difficult to account for underlying confounding factors. Further research is required to address these limitations and provide more conclusive findings.

In conclusion, our findings suggest urinary pentosidine as a potential biomarker for muscle status and physical performance even in young individuals. Pending future validations, urinary pentosidine could prove worthwhile as a marker of glycation stress when assessing health in young men.

## Data Availability

Not applicable.

## Abbreviations

AGEs: Advanced glycation end products
FFMI: Fat-free mass index
TUG: Timed up and go test
FR: Functional reach
NTx: Urinary N-telopeptide of type I collagen
Cre: Creatinine
